# Exploring bias in mechanical engineering students' perceptions of classmates

**DOI:** 10.1371/journal.pone.0212477

**Published:** 2019-03-07

**Authors:** Shima Salehi, N. G. Holmes, Carl Wieman

**Affiliations:** 1 Graduate School of Education, Stanford University, Stanford, California, United States of America; 2 Laboratory of Atomic and Solid State Physics, Department of Physics, Cornell University, Ithaca, New York, United States of America; 3 Department of Physics, Stanford University, Stanford, California, United States of America; Indiana University Bloomington, UNITED STATES

## Abstract

Gender disparity in science, technology, engineering, and math (STEM) fields is an on-going challenge. Gender bias is one of the possible mechanisms leading to such disparities and has been extensively studied. Previous work showed that there was a gender bias in how students perceived the competence of their peers in undergraduate biology courses. We examined whether there was a similar gender bias in a mechanical engineering course. We conducted the study in two offerings of the course, which used different instructional practices. We found no gender bias in peer perceptions of competence in either of the offerings. However, we did see that the offerings’ different instructional practices affected aspects of classroom climate, including: the number of peers who were perceived to be particularly knowledgeable, the richness of the associated network of connections between students, students’ familiarity with each other, and their perceptions about the course environment. These results suggest that negative bias against female students in peer perception is not universal, either across institutions or across STEM fields, and that instructional methods may have an impact on classroom climate.

## Introduction

While gender disparity in science, technology, engineering, and math (STEM) fields has been studied extensively during the last decade [1–4], attempts to increase the representation of females in STEM disciplines through classroom interventions have led to mixed results [5]. Therefore, it is critical to evaluate the mechanisms contributing to these disparities in order to achieve persistent change [6]. There are multiple possible mechanisms for these gender disparities at the undergraduate level, including general societal perceptions and stereotypes, and local factors such as perceived classroom and departmental norms and practices [7]. The latter often reflect social cognitive factors such as implicit bias, stereotype threat, and others [8–15]. Implicit gender bias has been clearly demonstrated in the evaluation of the competence of musicians [16], and it is plausible that similar effects would be present in other male-majority fields. Such implicit bias in college classrooms can have a significant impact on the chilly climate [17] for women in male-majority fields such as physical sciences and engineering [18].

Grunspan et al. examined students' gender bias in their perception of their peers’ competence (“peer perception bias”) in an undergraduate biology course [19]. They found that male students preferentially selected their male classmates as more knowledgeable compared to female classmates, while female students showed no such bias. Even if a female and a male student had the same grade, the male student was more likely to be nominated as knowledgeable by other male students. Furthermore, they found that “celebrities”, students who were chosen by the most students as being particularly knowledgeable, were overwhelmingly male.

Such gender bias in peer perception, if found consistently across institutions and STEM fields, would have important implications for understanding gender disparities in STEM. Biased perceptions would contribute to the chilly climate of STEM courses for female students, make women less likely to pursue these fields, and make it significantly harder for them to thrive if they do pursue these fields [20, 21]. Furthermore, perceptions of one’s peers plays a major role in hiring and progression in a field [21, 22], arguably a greater role than any objective measure of knowledge or ability.

The Grunspan et al. result was particularly worrisome, because the study was conducted in undergraduate biology courses. Unlike many other STEM fields, biology courses (and the discipline in general) have slightly more female students than male students [23]. We shared the view of Grunspan et al. (p. 12) that STEM fields with lower female representation might exhibit even larger peer perception biases compared to biology, as previous work has shown that female enrollment in science courses is negatively correlated with implicit and explicit science-gender-biases [24]. The lower presence of women in a STEM field can be particularly consequential for implicit gender biases in that field according to the associative-propositional evaluation model [25, 26]. According to this model, implicit biases are the results of associative processes in memory. Therefore, a lack of repeated exposure to women in a field (that is, few female role models, instructors, or peers) can lead to a weaker association between women and that field, and thus more implicit biases toward women. On the other hand, it is possible that women in engineering may be perceived as particularly exceptional *because* they have persisted in the male-majority discipline [27, 28], which may counter gender biases.

To test the hypothesis that peer perception bias would be more pronounced in male-majority fields, we conducted a similar study to that of Grunspan et al., but in an introductory mechanical engineering course. We chose this course because it was the largest engineering course at our institution in a strongly male-majority engineering discipline. Of course, such biases in STEM are not isolated to female students and also exist for many other minority groups. However, in the present study, we only looked at gender because of the small number of students in the class from other minority groups.

Grunspan et al.’s study also presented the possibility that classroom environment or instructional practices might mitigate the effects of gender on students’ perceptions of their peers. They found that the network of nominations and the strength of the peer perception bias differed between courses with an all-male instructional team and a course that used random call and included a female instructor. Random call has been found to eliminate gender gaps in class volunteers [29], and female instructors can reduce the science-gender-biases of female students [30]. It is possible that these aspects of the classroom impact how comfortable students are with speaking up in class, which in turn might impact their nominations. This raises broader questions about how classroom environments or instructional practices affect students’ peer perceptions. Another variable to consider would be how familiar students are with their peers in general, which could be very different in courses that use lecture-based instruction versus active learning [31].

We collected our data in two sequential offerings of the same introductory mechanical engineering course at Stanford University. Although these offerings covered the same content, they had different instructors (both tenured faculty members) who used very different instructional practices. One offering used traditional lecture-based instruction and was taught by a male instructor. The second offering had extensive use of active learning, including interactive lectures and group activities [32], and was taught by a female instructor. The variations in the offerings were not experimentally designed by the authors, but rather predetermined by the departmental constraints of these two offerings. That said, these variations allowed us to not only examine the generalizability of potential peer perception bias in mechanical engineering courses, but also to examine variations in students’ perception of their peers in different classroom environments, as hypothesized by Grunspan et al. We also examined students’ perception of the classroom environment itself across these two offerings to probe the impact of the classroom environment or context on the biases and nomination networks.

### The research questions

We conducted this study with the following research questions:

1. *Peer perception bias in another institution & field*: Will the gender bias in peer perception observed in Grunspan et al.’s study also be observed at a different institution and in a different field? (RQ1)
2. *Understanding factors affecting peer perception*: Expanding on the Grunspan et al.’s study, what factors other than gender shape peer perception and peer interaction? (RQ2)

## Materials and methods

### Course offerings and participants

The study was conducted in the fall and spring quarter offerings of the introductory mechanical engineering course. This course is a requirement for mechanical engineering majors as well as six smaller (but still male-majority) engineering majors: aeronautics and astronautics, biomechanical engineering, civil engineering, product design, environmental systems engineering, and architectural design. It was also an elective course for other engineering majors. The percentage of bachelor’s degrees awarded to women in the respective disciplines in 2015–2016 in the US were: mechanical engineering 13.8%; aerospace: 14.3%; biomedical: 41.4; civil engineering 24.0%; environmental systems engineering 28.3%; and architectural design 32.7% [33]. At Stanford university, for the year of data collection for this study (2015–2016), the percentages of women declaring their major in the engineering disciplines for which we have data are: mechanical engineering 36%; bioengineering 43%; civil engineering 43%; and environmental system engineering 81% (13 out of 16 students) [34].

The spring quarter offering of the course (“traditional”) was taught by a male instructor in a stadium-seating lecture hall. The typical class session involved lecture from the instructor along with multiple questions posed to students for individual answers. The fall quarter offering was taught by a female instructor in a classroom with moveable chairs and tables that could be rearranged for group work (“interactive”). Students were divided into four fixed groups to work on group activities in class. Students also had two group projects carried out over the course, with different groups for each project. The typical class session involved some lecture from the instructor with multiple questions to students, but almost all the class sessions included in-class lab and group activities, which required students to interact with each other. Student grades for the Traditional offering were based only on homework and exams, while for the interactive offering, the group projects and class discussion counted for 25% of the grade, with homework and exams making up the remaining 75%. The number of students enrolled in each offering by gender and grade level is given in Table 1. Because of prerequisites, freshmen students could not enroll in the fall offering.

**Table 1:** Student characteristics for the two course offerings.

	Spring (Traditional)	Fall (Interactive)
*Number of students enrolled in the course*	81 (31 female, 50 male)	83 (43 female, 40 male)
*Number of students participating in the study*	42 (15 female, 27 male)	75 (40 female, 35 male)
*School year of students*	11 freshman, 37 sophomore, 23 junior and 10 senior students	35 sophomore, 40 junior, 7 senior, and 1 other

The data were collected through a survey given in class. Students participating in the study, therefore, were ones who attended class and completed the survey on the day the survey was conducted. The student characteristics for each offering are summarized in Table 1. While both offerings had about the same number of enrolled students (approximately 80), the probability of students attending the class on the data collection date was significantly higher for the interactive offering (*p* = 0.0001), and this was not different across genders (*p* = 0.330). The female-male ratio was also higher in the interactive offering, suggesting some selection bias in the offering in which students chose to enroll. This selection may have been influenced by the gender of the instructor and/or the different teaching practices, both of which were known to most students before they enrolled.

For both offerings, the survey was administered in a paper format during the first 10 minutes of the class session, in week nine of the ten-week quarter. Grunspan et al. administered the survey multiple times throughout the course, but the largest bias was consistently observed at the end of the course. To comply with instructors’ desire to minimize the disruption, we only conducted the survey once and did so at the end of the course to maximize the signal. The first page of the survey included a written consent form for students to read and sign. Before starting the survey, the researcher summarized the content of the consent form including participants’ rights and privacy, instructed students to read the form carefully, and, if willing to participate in the study, sign it. For both offerings, the survey included the following parts:

1. A list of students enrolled in the class, along with a prompt to nominate the students from the list who they thought were “particularly strong in the course material.” The class list was in alphabetic order by last name for half of the surveys and in reverse alphabetic order for the other half to reduce the impact of the list sequence on the nomination pattern. We decided not to use a random list, as it would have made finding a particular name on the list much more difficult and time consuming for students. We did not say whether students could nominate themselves.
2. Two multiple-choice questions asking students about their familiarity with their classmates:
  - "What percentage of the names on page 2 did you recognize?"
  - "How many other students in the class have you discussed course material with (in or out of class)?"
3. Demographic questions, which asked for students’ name, ID number, gender, age, and their declared/intended major. Gender and age were multiple-choice questions, and the rest were open-response.
4. Four questions about students’ perception of the course learning environment taken in their entirety from a validated survey [35]. These questions asked the student to rate the following statements about the course environment from strongly disagree (1) to strongly agree (5), with question d) used to check for possible overall differences between the two offerings with regard to the students’ perceptions about diversity issues in general:
  - Students in the class try to help one another understand course material (e.g. sharing lecture notes when absent).
  - Students in the class consider themselves as part of a community.
  - I am comfortable making a comment or asking a question during class discussions.
  - Stanford University demonstrates a strong institutional commitment to diversity.

In the interactive offering, we also asked students to name the classmates with whom they interacted the most and to name their team members for the two class projects. For both offerings, the instructors provided the research team with all students’ course grades at the end of the quarter. The course grades were compared across genders and across participating and non-participating students (that is, those who attended and did not attend class on the data collection date). The Institutional Review Board of Stanford University approved this study under IRB protocol number 37719.

### Analysis

#### Probability of attending the class on the study date

We used logistic regression to compare the probability of students attending the class on the date of data collection for female and male students in each course offering.

#### Basic nomination patterns

We used linear multivariable regression to predict the number of nominations made by each student based on their gender, grade, and the course offering. We also used linear multivariable regression to predict the number of nominations received by each student based on their gender, grade, offering, and whether they participated in the study. It should be noted that nominators could only be the students participating in the study. However, nominees could be both from students participating or not participating in the study. Therefore, in analyzing the number of nominations received by each student, we also controlled for whether or not the nominee had participated in the study, by including a variable of “participated in the study” (no = 0, yes = 1) in the regression analysis. We controlled for participation because students who participated in the study were the ones who attended the class session on the study date, and probably the ones who more frequently attended class sessions in general. Therefore, these students are more likely to be better known by their peers, and therefore more likely to receive nominations from them.

#### Course grade

Course grades were in percentage points earned out of 100. We used the nonparametric Mann-Whitney U test (course grades were not normally distributed across genders) for each course offering to compare male and female students’ grades. We also used the nonparametric Mann-Whitney U test to compare the course grades of students who did and did not participate in the surveys to identify possible biases in our data collection.

#### ERGM network model for peer perception

To analyze the relationship between nominee and nominator gender, we used exponential random graph modeling [36] (ERGM), as used by Grunspan et al. ERGM estimates the log-odds of a link between two nodes and can be interpreted in a similar way as a logistic regression. In this case, each link represents a student nominating another student, where each student is a node. The estimates are based on numerical and categorical characteristics of each of the two nodes, as well as variation of these characteristics between each node. We used the ERGM method to analyze the nomination network to test whether, given the same method of analysis, the gender bias observed in the Grunspan et al. study exists in this study.

Similar to Grunspan et al.’s analysis, the following variables were included in the ERGM analysis of both courses:

1. Intercept
2. Mutuality of a nomination
3. Nominee and nominator being female [female-female bias]
4. Nominee and nominator being male [male-male bias]
5. Nominator being female
6. Nominee has no other nomination other than the one from this nominator [0-indegree]
7. Grade of nominee
8. Nominator and nominee being in the same discussion group (This was only included for the analysis of interactive offering, as there was no discussion group or lab in the traditional offering).

Grunspan et al. also had a measure of each student’s outspokenness based on teacher ratings. We did not collect this measure, as such ratings are likely to be subjective and may carry with them additional implicit bias [6].

For both offerings, we used Markov chain Monte Carlo (MCMC) diagnostics to check for mis-specification of ERGM models. ERGM passed these diagnostic tests, which indicates that the ERGM simulated networks are good representations of the observed social networks for both offerings (Figuress A, B, C and D in S1 File) [37, 38].

It has been shown that ERGM may lead to inaccurate and inconsistent estimates, in particular for sparse networks [39]. We, therefore, also analyzed the data using Logistic, Poisson, and linear regression; and compared the results to those of the ERGM (Tables C, D and E in S1 File). These other analyses gave similar conclusions as for the ERGM.

#### Celebrities

As in Grunspan et al.’s study [19], we also examined the gender and grade of “celebrities”, students who were chosen by the most students as being particularly knowledgeable. Celebrities of each offering were students whose number of received nominations was among the top quantile in that offering.

#### Self-nomination

We used logistic regression to compare the probability of self-nominations by gender in each offering.

#### Familiarity

We used Fisher’s exact test to compare the two offerings according to a) the number of students that each student indicated recognizing from the list and b) the number of students that each student indicated interacting with during the course.

#### Perception of the learning environment

We used nonparametric Mann-Whitney U tests to compare students’ responses to each of the Likert-scale questions about the learning environments between the two courses.

## Results

We first present an overall summary of the nomination patterns and student grades in the two course offerings. Then we present our findings regarding our two research questions: RQ1) peer perception bias in another institution and field, and RQ2) understanding factors affecting peer perception.

### Basic nomination patterns across the two offerings

**Fig 1:**
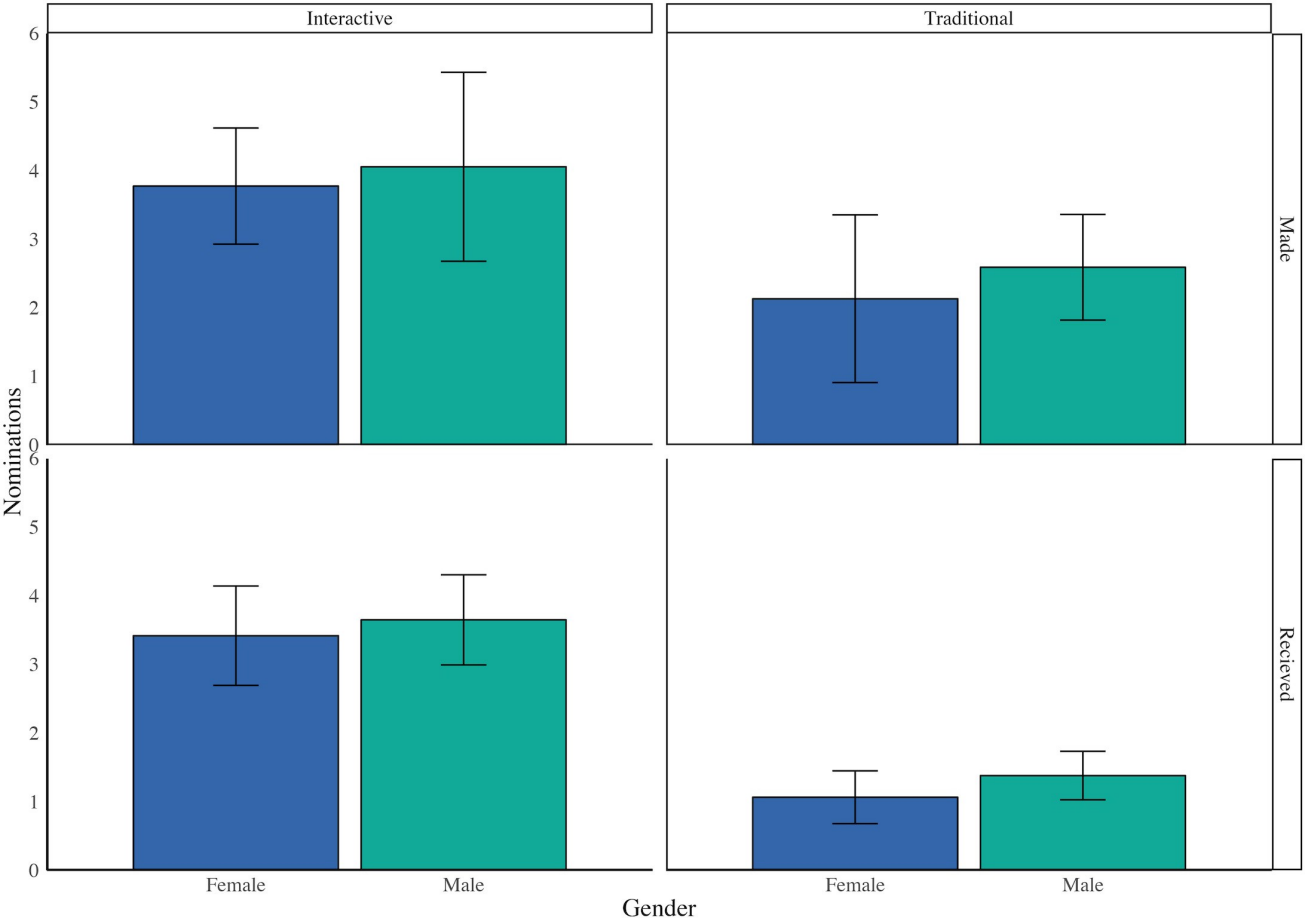
Average number of nominations made and received by each student across genders and course offerings. The error bars represent the 95% confidence intervals. The average number of nominations made and received is not the same for each category because the students who did not submit the survey could receive a nomination but could not make a nomination.

The breakdown of nominations between genders and offerings is shown in Fig 1. Students in the interactive offering, on average, made 3.92 nominations and received 3.54 nominations. Students in the traditional offering, on average, made 2.43 nominations and received 1.26 nominations. Linear multivariable regression analysis of the number of nominations made and received, controlling for students’ grade and gender, shows that the average number of nominations made and received by students in the interactive offering was significantly higher than in the traditional offering (difference between offerings in nominations made: *t*(113) = 2.70, *p* = 0.008; difference between offerings in nominations received: *t*(159) = 6.06, *p* < 0.0001). The details of these regression analyses are included in Tables A and B in S1 File. In this and the subsequent analysis, we have excluded self-nominations as in [19]. The pattern of self-nominations is discussed below separately.

**Fig 2:**
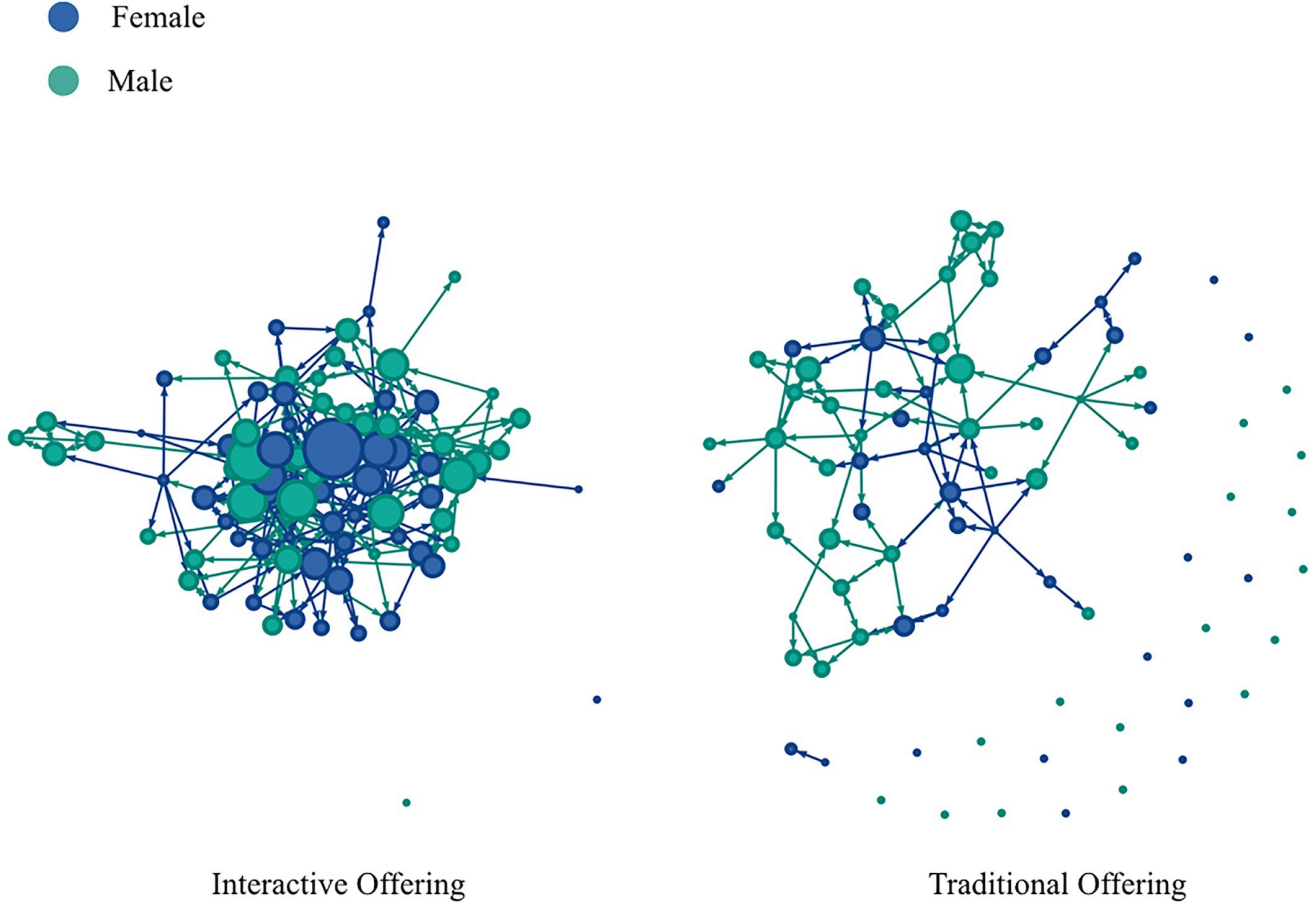
Network of students’ nominations for both offerings. Nodes represent students; links going out of each node represent nominations made by that student, and links going in to each node represent nominations received by the student. The size of each node represents the number of nominations received by that student.

The graphs for the nomination networks for the two offerings are shown in Fig 2. This illustrates the differences in the nomination patterns in the two offerings, with more overall nominations made and received in the interactive offering. It also shows that, in the interactive offering, the network of nominations was more interconnected compared to the traditional offering, and fewer students made no nominations or received none.

### Students’ grades

In the traditional offering, the median grade of female students was 5.5% lower than the median grade of male students (Mdn_male_ = 87.4, Mdn_female_ = 81.9, U = 571.5, *p* = 0.049), but in the interactive offering there was no difference (Mdn_male_ = 89.13, Mdn_female_ = 89.93, U = 874.5, *p* = 0.899).

For both offerings, there was a difference in grades between students participating and not participating in the survey. In the traditional offering, this difference was significant and the median score of students who participated was 13.4 percent higher than the median score of nonparticipants (Mdn_participating_ = 90.9, Mdn_Notparticipating_ = 77.5, *U* = 396.5, *p* <0.0001); in the interactive offering, the difference was 4.5 percent and marginally significant (Mdn_participating_ = 89.99, Mdn_Notparticipating_ = 85.46, *U* = 184, *p* = 0.074). As the attendance, and hence participation rate, for the traditional offering was substantially lower than for the interactive offering, it is possible that this differentially affected the nomination pattern of the students between the offerings.

### RQ1: Peer perception bias in a different institution and field

#### ERGM network model for peer perception

Table 2 shows the summary of the ERGM models for each offering. For both offerings, the gender of the nominator did not affect the probability of a student receiving a nomination (traditional: *p*_*gender*_ = 0.639; interactive: *p*_*gender*_ = 0.857); however, the grade of the nominee had a small but statistically significant effect on the probability of a student receiving a nomination (traditional: *p*_*grade*_ = 0.002; interactive: *p*_*grade*_ = 0.009). In looking at the dependence of nominations on the gender of the nominee, there was no significant male-male bias (traditional: *p* = 0.524, interactive: *p* = 0.223) or female-female bias (traditional: *p* = 0.161; interactive: *p* = 0.823) in the nominations received in either offering. This means that, controlling for the grade of the nominee, network structure, and student familiarity, neither female nor male students showed any gender preference in their nominations. This was true for both course offerings. These results are different from Grunspan et al.’s findings that male students were more likely to nominate their male peers than female peers.

**Table 2:** Coefficients from exponential random graph modeling (ERGM) for both offerings, with standard errors in parentheses.

Coefficient Name	Spring (Traditional)	Fall(Interactive)
*Intercept*	-6.62 (0.853)***	-6.038 (0.946)***
*Mutuality*	3.429 (0.530)***	3.033 (0.203)***
*Grade of nominee*	0.028 (0.009)**	0.027 (0.010)**
*0-indegree*	1.220 (0.631) **•**	0.736 (0.676)
*Female nominator*	-0.198 (0.421)	0.038 (0.214)
*Female-female bias*	0.471 (0.336)	0.035 (0.157)
*Male-male bias*	0.160 (0.251)	0.196 (0.160)
*Homophily in discussion group*	NA	0.361 (0.102)***

Significance codes:

p < 0.001 ***,

0.001< p < 0.01 **,

0.01 < p < 0.05 *,

0.05 < p < 0.1•.

Neither female nor male students show gender preferences in their nominations (lines 6 and 7).

To address limitations of ERGM, we checked the findings of the ERGM analysis three ways. First, by using logistic regression to predict the probability of a student being nominated. Second and third, by using multivariable linear and Poisson regressions to predict the number of nominations received by each student. In these three regression models, the predictors were: gender of nominee, gender of nominator, and course grade of nominee (Tables C, D and in S1 File). The addition of an interaction between gender of nominee and nominator in either of these models did not improve the model fit significantly, and when included, was either insignificant or marginally significant. Therefore, in these results we also did not find evidence for significant male-male bias in peer perception. Although the sample size in this study is much smaller than in Grunspan et al., an analysis of our statistical power, as shown in Table F in S1 File, indicates that if there was a bias in either course offering of the size observed in Grunspan et al.’s study, we could have rejected the null hypothesis that there is no bias with a *p-*value of 0.02 or smaller.

#### Celebrity status and course performance

**Fig 3:**
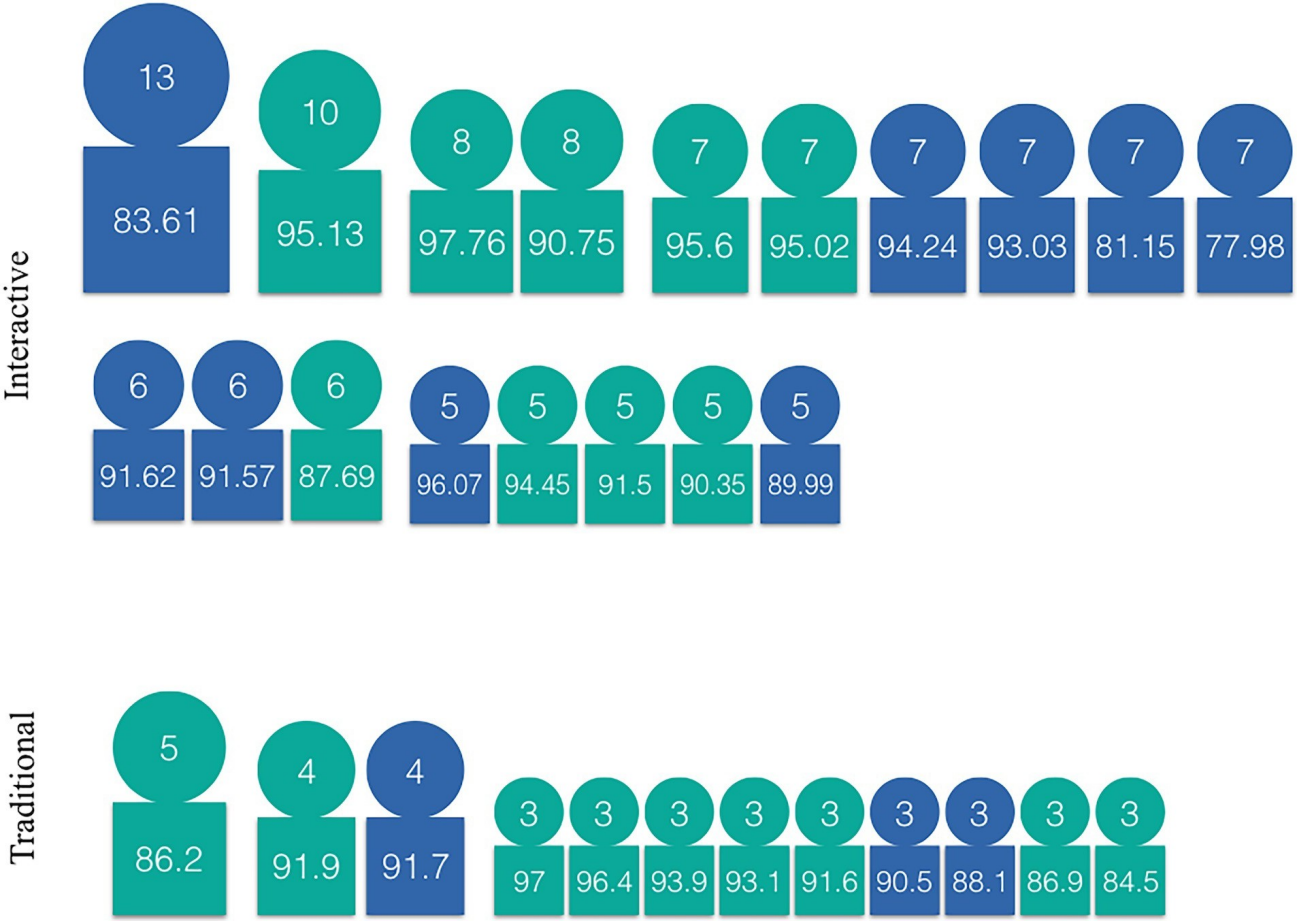
Students receiving the most nominations in the two offerings, by number of received nominations and course grades. Green students are male and blue are female. Recall the ratio of the number of male to female students was 1.8 for the traditional offering and 0.9 for the interactive offering.

Grunspan et al. also looked at the gender composition of students receiving exceptional numbers of nominations (“celebrities”). They found that the celebrities were predominantly male, the student with the most nominations in a class was always male, the highest rank for female celebrities was fourth, and, given the same grade, female students had lower celebrity rankings than their male peers. In our study, as shown in Fig 3, the most nominated student in the traditional offering was male and the second was female with only one fewer nomination (5 vs. 4). In the interactive offering, the most nominated student was female. Overall, there is no obvious pattern of gender preference in celebrity status. Also, as can be seen in Fig 3, in both offerings, the student with the highest grade was ranked third. We conclude that the prerequisite for becoming a celebrity in these courses was neither the highest grade nor being a male, in clear contrast with Grunspan et al.’s findings.

#### Self-nomination

An unforeseen finding in our data was that of self-nomination. As discussed above, we (and Grunspan et al.) excluded self-nominations from the analyses above because we were interested in students’ perceptions of their *peers*. We found, however, that male students nominated themselves with marginally higher probability regardless of the course offering (*p* = 0.092). This result is consistent with previous reports of male students having higher self-concept [40] and male scholars being more likely to cite themselves [41]. Approximately a third of participating male students (8 out of 27 in the traditional offering and 12 out of 35 in the interactive offering) nominated themselves as being highly knowledgeable. However, for female students, these percentages were 6% (1 out of 15) and 18% (7 out of 40), for the traditional and interactive offerings, respectively. In the interactive offering, students of both genders nominated themselves with higher probability, but the difference between offerings was much larger for female students. Due to the small number of self-nominations, particularly for female students, these differences were not statistically significant (*p* = 0.467), however. Nonetheless, this result suggests that looking at self-nominations of competence might be a novel way to explore how female and male students’ self-confidence is influenced by instructional practices.

### RQ2: Understanding factors affecting peer perception

To better understand the distinctions in the impact of the different offerings, we examined differences between the offerings in the students’ familiarity with their peers and, to a lesser extent, their perceptions of the class environment.

#### Students’ familiarity with their peers

In the interactive offering, we asked students to name the peers they worked with most often, as well as their project team members. The majority of students’ nominations (64%, SE = 4%) were from these proximity circles, and this percentage was not different across students’ gender (U = 554, *p* = 0.86; Mdn_female_ = 0.33, Mdn_male_ = 0.39). This result is consistent with the ERGM result that mutuality was the strongest factor influencing the probability of students’ nominating a peer.

We determined students’ overall familiarity with their peers through their responses to two questions on the paper survey that probed: 1) What percentage of the names on the class list they recognized, and 2) How many other students in the class they discussed the material with (in or out of class). The responses to these two items, shown in the Tables 3 and 4, indicate that, not surprisingly, students in the interactive offering knew significantly more of their classmates (Fisher’s exact test: *p* < 0.0001), and interacted with significantly more of them (Fisher’s exact test: *p* = 0.003). In the traditional offering, 32.5% of the participants replied that they recognized 0% of their classmates’ names. In the interactive offering, in contrast, there was no student who did not recognize any of their classmates’ names. In the traditional offering, only 24.4% of students reported that they had discussed the course material with more than 5 of their classmates, while, in the interactive offering, this fraction was 45.3%. The difference in the students’ familiarity and interactions with their classmates can explain the higher number of nominations made in the interactive offering compared to the traditional offering.

**Table 3:** Percentage of students’ names participants recognized across the two offerings.

	Percentage of classmates recognized	
	No classmate recognized	Some classmate recognized
*Traditional*	32.5%	67.5%	
*Interactive*	0%	100%

**Table 4:** Number of classmates with whom students discussed course material.

	Number of classmates with whom students discussed course material	
	Less than 5	5 or more than 5
*Traditional*	75.6%	24.4%
*Interactive*	54.7%	45.3%

#### Students’ perception of the learning environment

**Fig 4:**
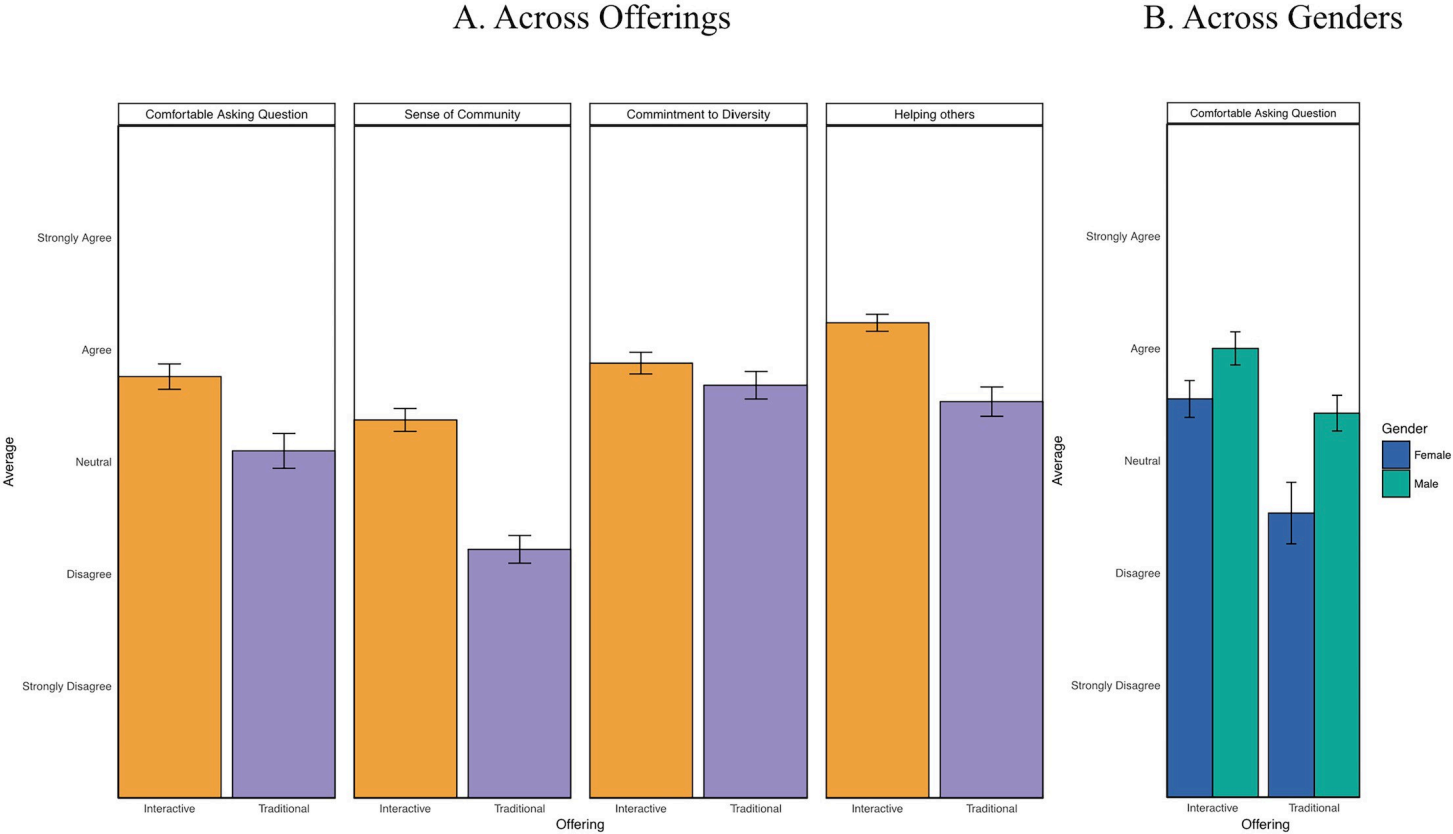
A. Average responses to the perception questions. B. Responses to “Comfortable asking questions” item broken down by gender and course offering. This was the only item that showed a significant gender difference. The error bars represent standard errors.

Grunspan et al. observed a suggestive pattern that the course learning environment might have influenced students’ peer perception bias. To explore this issue, we asked students about their perception of the course learning environment explicitly [32]. We saw a difference in students’ perception of the learning environments as shown in Fig 4, with students of both genders indicating more positive perceptions of the interactive class environment. They found it to be a more supportive environment (*U* = 2237, *p* < 0.0001, Hodges-Lehmann estimator Effect size = 0.42), with a higher sense of community (*U* = 2526.5, *p* < 0.0001, Hodges-Lehmann estimator Effect size = 0.56), and were more comfortable asking questions (*U* = 2064, *p* = 0.001, Hodges-Lehmann estimator Effect size = 0.30). Also, female students were significantly less comfortable asking questions in the traditional offering than male students (*U = 104*, *p = 0*.*009*, Hodges-Lehmann estimator Effect size = *0*.*29)*, while this difference was marginal in the interactive offering (*U* = 530, *p* = 0.059, Hodges-Lehmann estimator Effect size = *0*.*22*). Students in the two offerings did not differ in their perception of the university’s commitment to diversity (*U* = 1764.5, *p* = 0.160), suggesting that their responses to the other questions were determined by the specific class environment, rather than general perceptions about the institution and their fellow Stanford students as a whole.

Future work should evaluate whether these patterns reproduce and how they moderate students’ peer perception in courses, while controlling for students’ selection biases and effects of instructor gender. This will clarify whether interactive instructional practices lead to classroom environments that encourage a greater sense of belonging and a supportive community. Other work [32] has shown indication of such effects.

## Discussion

To study a potential mechanism for gender disparity in STEM, we examined whether students in a mechanical engineering course showed gender bias in peer perceptions as observed by Grunspan et al. [19] in biology classes. Grunspan et al. observed a bias against female students by their male classmates in perceived knowledge of the subject. We conducted the study in two offerings of an introductory mechanical engineering course to test the generalizability of these results in another institution and a more male-majority field.

In contrast to the previous study in introductory biology, and our own hypothesis, we saw no bias against female students. These findings suggest that the gender bias reported by Grunspan et al. is not necessarily generalizable to different STEM fields and/or different institutions. The different findings across the two studies calls for further investigation to understand potential differences in gender biases in student perceptions of their peers across different STEM fields and different institutions.

In both the interactive and traditional course offerings, we saw that the probability of receiving a nomination was mainly associated with students’ familiarity with their peers and, to a lesser extent, the grade of the potential nominee. In the interactive offering, we also asked students to list friends and project members. We observed that more than 60% of nominations made by each nominator were from their listed friends and project members. Students were more familiar with each other in the interactive offering, made more nominations, and correspondingly had a more interconnected network.

The different instructional approaches used in the two offerings of the course probably impacted the different levels of familiarity and associated number of nominations. We noted a number of other intriguing differences between the two offerings of the course, which we speculate might be associated with the differences in the instructional practices. Students perceived the interactive course to be a more comfortable and supportive community. Our study does not allow us to draw conclusions as to the connection between these perceptions and the teaching methods used because of the likely presence of selection bias between the two offerings, as well as the difference in instructor gender. However, these initial observations can inform future studies to investigate the effects of instructional practices on students’ perception of the learning environments and possible impacts on peer perceptions.

A limitation of our study is that a number of students did not attend the class on the date of data collection, and so did not nominate their peers. This number was larger in the traditional offering. However, as mentioned in the results section, male and female students were equally likely to not participate in the study, so these missing students are unlikely to affect the determination of gender bias.

Our study differs from that of Grunspan et al. in a number of ways that may have contributed to the differing results. One factor is class size. The Grunspan et al.’s study used three courses, each with hundreds of students. We had 81 and 83 students in the two offerings studied. As discussed in the power analysis in S1 File, we had sufficient statistical power to observe bias of the reported size, but there could be other factors that depend on class size. For example, class size could also have contributed to the results through the impact on students’ familiarity with their classmates. It is plausible that this could result in different nomination patterns between large and small classes. In a small class, it is relatively easy to get to know a larger fraction of fellow students in the classroom setting, and hence have better knowledge of their competence compared to what is possible in a large class. This will mean that the nomination patterns of students in large classes will be more sensitive to any gender imbalances to their familiarity circles.

Another difference between the two studies is the level of students. In our study, the students were more advanced, spanning all four years, and it is possible this influenced the extent of the peer perception bias. We cannot rule out the possibility that as the students advance in STEM fields, biases are reduced because students are more integrated into the culture [21, 22]. The two different institutions are also rather different, one being relatively small and highly selective, while the other is a much larger public institution, and this may result in differences in the student populations.

The other major difference between the two studies was the academic fields under study: biology compared to mechanical engineering. Biology as a field has stronger representation of women than mechanical engineering. We, as well as Grunspan et al. (p. 12), initially hypothesized that low female representation in male-majority STEM fields might lead to a larger peer perception bias in these fields compared to biology. However, cultural norms, characteristics of the institution, instructional practices, and role models in the field or in the institution might have stronger effects on gender biases in a specific discipline than the mere gender ratio. The possible impacts of these local issues and how they interact with the broader image of the field to influence gender bias warrant further study.

Understanding factors that contribute to gender biases and resulting variations in gender biases across different STEM fields or institutions is critically important, as both overestimation and underestimation of gender biases across fields are harmful. Overestimation of gender biases can send discouraging but untrue messages to female students considering entering those fields, while underestimation can inhibit the efforts needed to address such biases where they exist. In addition, the more nuanced understanding of gender biases can inform more targeted efforts for each field to overcome these biases. Grunspan et al. concluded that “our work implies that the chilly environment for women may not be going away any time soon” (p. 13 of [19]). Our study suggests that the environment may not be as chilly as they concluded.

## Supporting information

S1 File Supporting analysis. Additional regression analyses to ERGM, and power analysis for ERGM.
(DOCX)

S2 File Data for the traditional offering. (CSV)

S3 File Data for the interactive offering. (CSV)

S4 File Link data for the traditional offering used in ERGM analysis. (CSV)

S5 File Node data for the traditional offering used in ERGM analysis. (CSV)

S6 File Link data for the interactive offering used in ERGM analysis. (CSV)

S7 File Node data for the interactive offering used in ERGM analysis. (CSV)

S8 File R code for analysis. (RMD)

## References

[1] National Science Foundation, Arlington, Va. Women, minorities, and persons with disabilities in science and engineering: 1996. National Science Foundation; 1996.

[2] Larivière V, Ni C, Gingras Y, Cronin B, Sugimoto CR. Bibliometrics: Global gender disparities in science. Nature News. 2013 12 12;504(7479):211. 10.1038/504211a 24350369

[3] Eagly AH, Mladinic A. Are people prejudiced against women? Some answers from research on attitudes, gender stereotypes, and judgments of competence. European review of social psychology. 1994 1 1;5(1):1–35.

[4] Hill C, Corbett C, St Rose A. Why so few? Women in science, technology, engineering, and mathematics. American Association of University Women 1111 Sixteenth Street NW, Washington, DC 20036; 2010.

[5] Madsen A, McKagan SB, Sayre EC. Gender gap on concept inventories in physics: What is consistent, what is inconsistent, and what factors influence the gap?. Physical Review Special Topics-Physics Education Research. 2013 11 18;9(2):020121.

[6] Eddy SL, Brownell SE. Beneath the numbers: A review of gender disparities in undergraduate education across science, technology, engineering, and math disciplines. Physical Review Physics Education Research. 2016 8 1;12(2):020106.

[7] Kost LE, Pollock SJ, Finkelstein ND. Unpacking gender differences in students’ perceived experiences in introductory physics InAIP conference proceedings 2009 11 5 (Vol. 1179, No. 1, pp. 177–180). AIP.

[8] Greenwald AG, Krieger LH. Implicit bias: Scientific foundations. California Law Review. 2006 7 1;94(4):945–67.

[9] Bohnet I. What works: Gender equality by design. Cambridge, MA: Belknap Press of Harvard University Press; 2016 3 8.

[10] Moss-Racusin CA, Dovidio JF, Brescoll VL, Graham MJ, Handelsman J. Science faculty’s subtle gender biases favor male students. Proceedings of the National Academy of Sciences. 2012 10 9;109(41):16474–9. 10.1073/pnas.1211286109 PMC3478626 22988126

[11] Reuben E, Sapienza P, Zingales L. How stereotypes impair women’s careers in science. Proceedings of the National Academy of Sciences. 2014 3 5:201314788. 10.1073/pnas.1314788111 PMC3970474 24616490

[12] Spencer SJ, Steele CM, Quinn DM. Stereotype threat and women's math performance. Journal of experimental social psychology. 1999 1 1;35(1):4–28.

[13] Proudfoot D, Kay AC, Koval CZ. A gender bias in the attribution of creativity: Archival and experimental evidence for the perceived association between masculinity and creative thinking. Psychological Science. 2015 11;26(11):1751–61. 10.1177/0956797615598739 26386015

[14] Ceci SJ, Williams WM, Sumner RA, DeFraine WC. Do subtle cues about belongingness constrain women's career choices?. Psychological Inquiry. 2011 10 1;22(4):255–8. 10.1080/1047840X.2011.619112 23136463 PMC3489489

[15] Kelly AM. Social cognitive perspective of gender disparities in undergraduate physics. Physical Review Physics Education Research. 2016 8 1;12(2):020116.

[16] Goldin C, Rouse C. Orchestrating impartiality: The impact of" blind" auditions on female musicians. American economic review. 2000 9;90(4):715–41.

[17] Sandler BR, Hall RM. The classroom climate: A chilly one for women. Washington, DC: Association of American Colleges 1982.

[18] Gunter R, Stambach A. Differences in men and women scientists’ perception of workplace climate. Journal of Women and Minorities in Science and Engineering. 2005;11(1).

[19] Grunspan DZ, Eddy SL, Brownell SE, Wiggins BL, Crowe AJ, Goodreau SM. Males under-estimate academic performance of their female peers in undergraduate biology classrooms. PloS one. 2016 2 10;11(2):e0148405 10.1371/journal.pone.0148405 26863320 PMC4749286

[20] Laube H, Massoni K, Sprague J, Ferber AL. The impact of gender on the evaluation of teaching: What we know and what we can do. NWSA Journal. 2007 10 1:87–104.

[21] National Research Council. Gender differences at critical transitions in the careers of science, engineering, and mathematics faculty. National Academies Press; 2010 7 18.

[22] Cole MS, Feild HS, Giles WF. Interaction of recruiter and applicant gender in resume evaluation: a field study. Sex Roles. 2004 11 1;51(9–10):597–608.

[23] Wiens DJ, Depping DJ, Wallerich SR, Van Laar ES, Juhl AL. Gender matters. Journal of College Science Teaching. 2003 9 1;33(1):32.

[24] Miller DI, Eagly AH, Linn MC. Women’s representation in science predicts national gender-science stereotypes: Evidence from 66 nations. Journal of Educational Psychology. 2015 8;107(3):631.

[25] Gawronski B, Bodenhausen GV. Associative and propositional processes in evaluation: an integrative review of implicit and explicit attitude change. Psychological bulletin. 2006 9;132(5):692 10.1037/0033-2909.132.5.692 16910748

[26] Gawronski B, Bodenhausen GV. The associative–propositional evaluation model: Theory, evidence, and open questions InAdvances in experimental social psychology 2011 1 1 (Vol. 44, pp. 59–127). Academic Press.

[27] Dryburgh H. Work hard, play hard: Women and professionalization in engineering—adapting to the culture. Gender & Society. 1999 10;13(5):664–82.

[28] Ghiasi G, Larivière V, Sugimoto CR. On the compliance of women engineers with a gendered scientific system. PloS one. 2015 12 30;10(12):e0145931 10.1371/journal.pone.0145931 26716831 PMC4696668

[29] Eddy SL, Brownell SE, Wenderoth MP. Gender gaps in achievement and participation in multiple introductory biology classrooms. CBE—Life Sciences Education. 2014 9;13(3):478–92. 10.1187/cbe.13-10-0204 25185231 PMC4152209

[30] Young DM, Rudman LA, Buettner HM, McLean MC. The influence of female role models on women’s implicit science cognitions. Psychology of Women Quarterly. 2013 9;37(3):283–92.

[31] Brewe E, Kramer LH, O’Brien GE. Changing participation through formation of student learning communities. InAIP Conference Proceedings 2010 10 24 (Vol. 1289, No. 1, pp. 85–88). AIP.

[32] Prince M. Does active learning work? A review of the research. Journal of engineering education. 2004 7;93(3):223–31.

[33] Yoder B. Engineering by the numbers. ASEE (American Society of Engineering Educators).

[34] School of Engineering Enrollment 2015–16 [Cited 15 August 2018]. Available from: https://registrar.stanford.edu/everyone/enrollment-statistics/enrollment-statistics-2015-16/school-engineering-enrollment-2015-16

[35] Ballen CJ, Wieman C, Salehi S, Searle J, Zamudio KR. Active learning improves diversity in undergraduate science. CBE-Life Sciences Education (In review). 2017 9. 10.1187/cbe.16-12-0344 PMC5749958 29054921

[36] Robins G, Pattison P, Kalish Y, Lusher D. An introduction to exponential random graph (p*) models for social networks. Social networks. 2007 5 1;29(2):173–91.

[37] Handcock MS, Hunter DR, Butts CT, Goodreau SM, Morris M. statnet: Software tools for the representation, visualization, analysis and simulation of network data. Journal of statistical software. 2008;24(1):1548 MCMC diagnostics examines whether simulated networks converge. The model estimates are tested to ensure they converge to a constant value and the residual of the estimates should have a bell-shaped distribution around zero. 18618019 10.18637/jss.v024.i01 PMC2447931

[38] Morris M, Handcock MS, Butts CT, Hunter DR, Goodreau SM, de-Moll SB, Krivitsky PN. Exponential random graph model (ERGMs) using statnet [Internet]. Sunbelt US 2016 [Cited 15 February 2017]. Available from: https://statnet.org/trac/raw-attachment/wiki/Sunbelt2016/ergm_tutorial.html#References.

[39] Chandrasekhar AG, Jackson MO. Tractable and consistent random graph models. National Bureau of Economic Research; 2014 7 7, and personal communications with the authors.

[40] Cooper KM, Krieg A, Brownell SE. Who perceives they are smarter? Exploring the influence of student characteristics on student academic self-concept in physiology. Advances in physiology education. 2018 6 1;42(2):200–8. 10.1152/advan.00085.2017 29616569

[41] King MM, Bergstrom CT, Correll SJ, Jacquet J, West JD. Men set their own cites high: Gender and self-citation across fields and over time. arXiv preprint arXiv:1607.00376. 2016 Jun 30.

