# Supplementary material for: Exploring bias in mechanical engineering students' perceptions of classmates
Source: PLoS One. 2019 Mar 7;14(3):e0212477. doi: 10.1371/journal.pone.0212477 (PMC6405061; doi:10.1371/journal.pone.0212477)
Supplement: S1 File — Additional regression analyses to ERGM, and power analysis for ERGM. (DOCX) [file pone.0212477.s001.docx]

# Supporting Information

## Basic nomination patterns across the two offerings

The following tables summarize the linear regression analyses for the average number of nominations made and received by each student. In addition to the results of the linear regression models shown, as a test of the sensitivity to the model assumptions, we also carried out similar analysis using Poisson regression models, and we found the same overall results.

**Table A. Coefficients from regression analyses for average nominations made.**

| Coefficient Name | Linear Regression |
| --- | --- |
| *Intercept* | 3.70 (0.44)  *p* < 0.0001*** |
| *Gender of nominator (Male=1)* | 0.48 (0.57)  *p* = 0.403 |
| *Normalized grade of nominator* | -0.48 (0.28)  *p* = 0.088 **•** |
| *Offering*  *(Interactive=1)* | 1.6 (0.59)  P = 0.008 ** |
| *AIC* | 597.30 |

**Table B. Coefficients from regression analyses for average nominations received.**

| Coefficient Name | Linear Regression |
| --- | --- |
| *Intercept* | 2.77 (0.40)  *p* < 0.0001*** |
| *Gender of nominee (Male=1)* | 0.22 (0.28)  *p* = 0.433 |
| *Participated in the study*  *(Yes=1)* | 0.62(0.36)  *p* = 0.092 **•** |
| *Normalized grade of nominee* | 0.42 (0.16)  *p* = 0.008 ** |
| *Offering*  *(Interactive=1)* | 1.85 (0.31)  *p* < 0.0001*** |
| *AIC* | 656.08 |

## Complementary regression Analysis

To further check the ERGM analysis, we also analyzed the nomination data using logistic, Poisson, and normal multivariable regression, and compared the results of each method. Logistic regression models the probability of receiving a nomination based on the predictor variables, with the assumption that the distribution of nominations is normal. Poisson regression models the log of the expected counts (in this case the number of received nominations) as a function of the predictor variables, with the assumption that the distribution of nominations received is Poisson. Normal multivariable regression models the number of nominations received by each nominee as a function of the predictor variables, with the assumption that the distribution of nominations is normal. For all the methods, the main model predictor variables (independent variables) were as follows:

1. Gender of nominee
2. Gender of nominator
3. Grade of nominee (centered around mean and divided by standard deviation)

Discussion group variables for matching the nominator and nominee, as included in the ERGM model, require a different structure of the data than these three regression methods allow, so were not included in the regression analyses. One significant limitation of the Poisson method is that it does not allow for normalizing the number of nominations to effectively control for the number of male and female students in the samples.

For all three methods, we tested whether adding interactions between the predictor variables would improve the fit of the model. None of the interaction terms significantly improved the model fit, including the interaction between the gender of nominee and nominator. When added to the model, the coefficient of the interaction between the gender of nominee and nominator, as reported below, was at largest marginally significant in some models, and insignificant in the others. Therefore, we do not find credible evidence for male-male peer perception bias in these analyses. The following three tables report the results of the logistic, Poisson, and linear regressions for the two offerings, including the (not significant) interaction term of particular interest, gender of nominee with gender of nominator.

**Table C. Coefficients from the logistic regression analyses for each offering.**

| Logistic Coefficient | Spring (Traditional) | Fall (Interactive) |
| --- | --- | --- |
| *Intercept* | -0.34 (0.39)  *p* = 0.384 | 2.06 (0.48)  *p* < 0.0001 *** |
| *Gender of nominee*  *(Male=1)* | -0.96 (0.53)  *p* = 0.073 **•** | -0.73 (0.62)  *p* = 0.238 |
| *Normalized grade of nominee* | 1.00 (0.23)  *p* < 0.0001 *** | 0.46 (0.19)  *p* = 0.017 * |
| *Gender of nominator*  *(Male=1)* | 0.15 (0.55)  *p* = 0.782 | -0.72 (0.61)  *p* = 0.242 |
| *Gender of nominee* x  *Gender of nominator* | 1.25 (0.72)  *p* = 0.085 **•** | 1.74 (0.91)  *p* = 0.056 **•** |

**Table D. Coefficients from the Poisson regression analyses for each offering.**

| Poisson Coefficient | Spring (Traditional) | Fall (Interactive) |
| --- | --- | --- |
| *Intercept* | -0.78 (0.27)  *p* = 0.004 ** | 0.60 (0.11)  *p* < 0.0001 *** |
| *Gender of nominee*  *(Male=1)* | -0.45 (0.36)  *p* = 0.208 | -0.04 (0.16)  *p* = 0.827 |
| *Normalized grade of nominee* | 0.56 (0.13)  *p* < 0.0001 *** | 0.17 (0.07)  *p* = 0.009 ** |
| *Gender of nominator*  *(Male=1)* | 0.31 (0.35)  *p* = 0.386 | -0.18 (0.17)  *p* = 0.284 |
| *Gender of nominee* x  *Gender of nominator* | 0.74 (0.45)  *p* = 0.099 **•** | 0.25 (0.23)  *p* = 0.294 |

**Table E. Coefficients from the linear regression analyses for each offering.**

| Linear Coefficient | Spring (Traditional) | Fall (Interactive) |
| --- | --- | --- |
| *Intercept* | 0.03 (0.007)  *p* < 0.0001 *** | 0.05 (0.006)  *p* < 0.0001*** |
| *Gender of nominee*  *(Male=1)* | -0.01 (0.009)  *p* = 0.265 | -0.001 (0.008)  *p* = 0.873 |
| *Normalized grade of nominee* | 0.01 (0.003)  p < 0.0001 *** | 0.007 (0.003)  *p* = 0.019* |
| *Gender of nominator*  *(Male=1)* | -0.007(0.01)  *p* = 0.458 | -0.002 (0.008)  *p* = 0.809 |
| *Gender of nominee* x  *Gender of nominator* | 0.02 (0.01)  *p* = 0.097 **•** | 0.01 (0.01)  *p* = 0.317 |

## Power Analysis

There is no conventional power analysis method for ERGM (personal communication with Matthew Jackson [36]). Therefore, we used a binomial model to analyze the statistical sensitivity of our samples to a bias of the size reported in Grunspan et al. As discussed in detail below, we started by assuming each class offering was a binomial sample distribution with a given fraction of male and female students, and each nomination made by a male corresponds to sampling this distribution. For each course offering, we then calculate the total number of male nominations made by male students that we would expect if there was no bias (N_unbias_), and then we repeat the calculation using a bias of the average size observed in Grunspan et al.’s study to predict the number of male-male nominations if there was bias (N_bias_). Finally, we use the standard P value calculations for binominal distributions to calculate the probability of observing N_bias_ with our sample sizes when the true mean of the sample is N_unbias_.

If in making a nomination, every male is unbiased and so equally likely to nominate a male as to nominate a female student; then the probability of a nomination from a male student to another male student (male-male nomination) is equal the fraction of the class that is male (50/81 and 40/83 in the traditional and interactive offerings respectively). Therefore, the overall number of male-male nominations in the offering is this unbiased probability times the total number of nominations made by male students (70 and 143). This gives expected values, N_unbias_, of 43 and 69 male-male nominations, respectively, in the two offerings. To find N_bias_ for comparison, we need to know how much the probability of a male nominating another male is enhanced when there is a bias, given the size of male-male nomination bias. To estimate the size of the bias, we used Grunspan et al.’s results reported in Fig.2 (p. 9 of [19]). In this figure, Grunspan et al. modeled the biased proportions of nominations to male students from other male students in a gender-balanced classroom. From this figure, we can then identify a bias factor, *b*, as the biased proportion of male-male nominations divided by 0.5, the value that would be obtained if there was no bias, as this is for the model of gender-balanced classes. That is,

$$b=\frac{p_{m-m}}{0.5}$$

where $p_{m-m}$is the biased proportions of nominations to male students from other male students.

Taking the end of the quarter values shown for the classes in Grunspan et al.’s Fig. 2 (p.9), this bias factor, b, is .63/.5 = 1.26 for the class with the largest bias and is .60/.5 =1.21 for the average across all classes. Multiplying our unbiased male-male nominations probability by the bias factor then gives the biased probability of a male-male nomination for each of our two offerings. Multiplying these biased probabilities by the overall number of male-male nominations in each offering gives the expected biased number of male-male nominations. Using the average value of b, 1.21, thus, N_bias_ equals 53, and 83 for the two offerings.

We then calculate the probability of being unable to distinguish between the expected biased and expected unbiased numbers of male-male nominations in our two samples, assuming binomial distributions, as shown in the bottom line of Table F. If there was a bias with the average size reported by Grunspan et al. then we could have rejected the null hypothesis (that of no bias in our sample) with a *p*-value of 0.02 and 0.01 for the two offerings.

**Table F. Binomial power analysis for our two offerings based on the average effect size, b, of 1.21 determined through Grunspan et al.’s Fig. 2. If we use b = 1.26, the largest bias reported for a single course, the P values are about 1/3 as large as shown for b_avg_.**

|  | Traditional | Interactive | |
| --- | --- | --- | --- |
| *Total number of nominations of males made by males, male-male, N* | *70* | *143* | |
| *Fraction of males in class, P_unbiased_* | *50/81* | *40/83* |  |
| *Expected number of male-male nominations if unbiased, N_unbias_ = N * P_unbiased_* | *43* | *69* |  |
| *P_biased_ (_avg_)= b (_avg_)* P_unbiased_* | *0.75* | *0.58* | |
| *Expected number of male-male nominations if biased, N_bias_ = N * P_biased (avg)_* | *53* | *83* |  |
| *P (male-male nominations* $\geq$ *N_bias_) in a binomial sample with the mean of N_unbias_* | *0.02* | *0.01* | |

Our statistical power is helped by the fact that, although we have smaller class sizes than in Grunspan et al.’ study, the average number of nominations made by each student in our study was higher, with 2.4 nominations per nominee for the traditional offering and 3.9 for the interactive offering, compared to 1.2 in Grunspan et al.’s study. This difference between the two studies is also reflected in the larger intercept for the ERGM models in this study compared to the intercepts for the ERGM models in Grunspan et al.’s study.

## Goodness of Fit


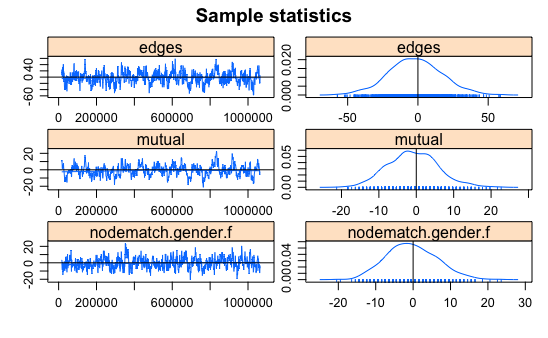


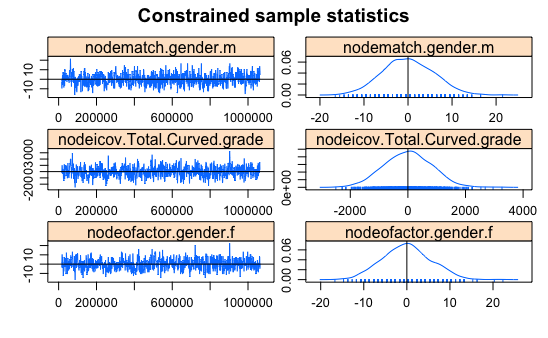


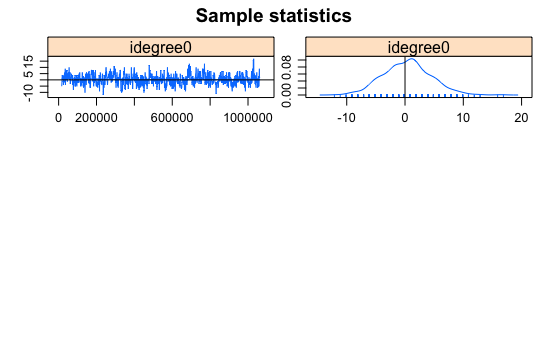


**Figure A. MCMC diagnostics for traditional spring offering.**


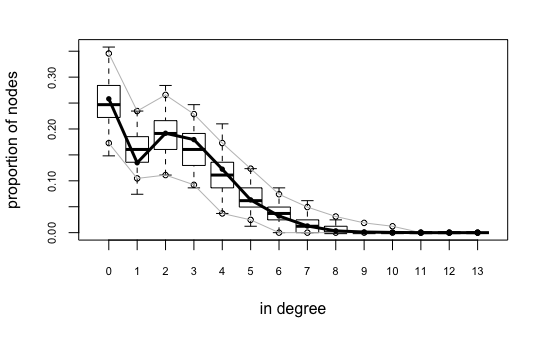

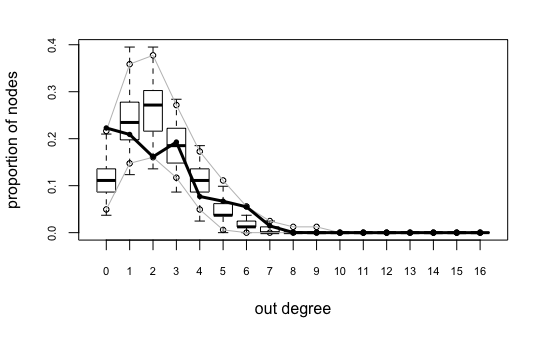

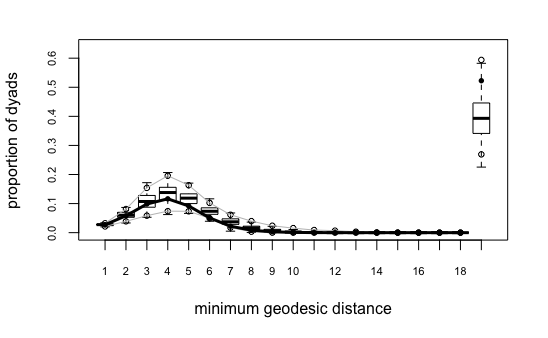

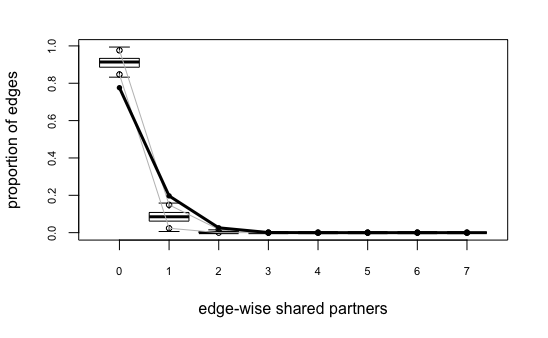


**Figure B. Goodness of fit plots for traditional spring offering.**


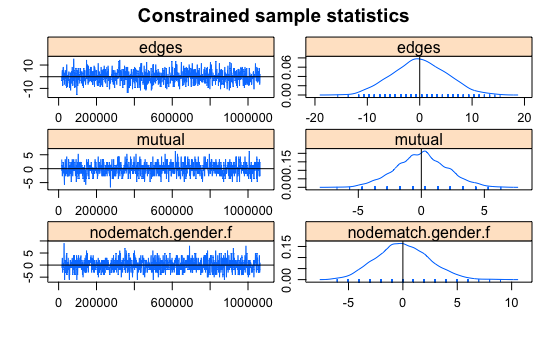

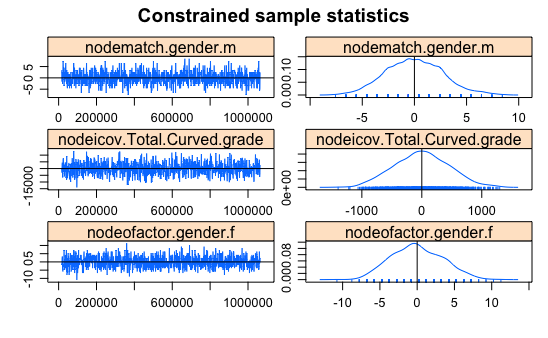


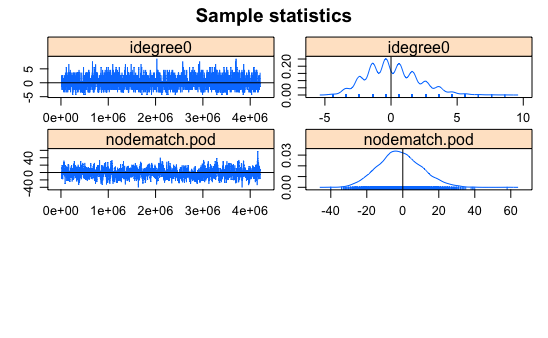
**Figure C. MCMC diagnostics for interactive fall offering.**


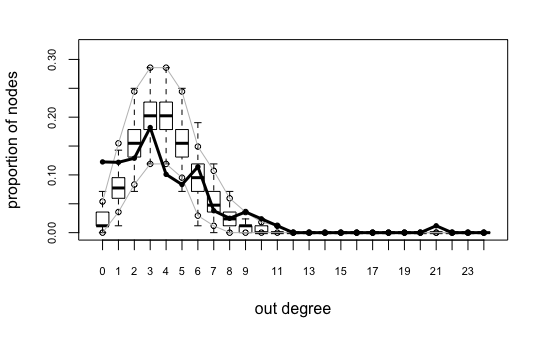

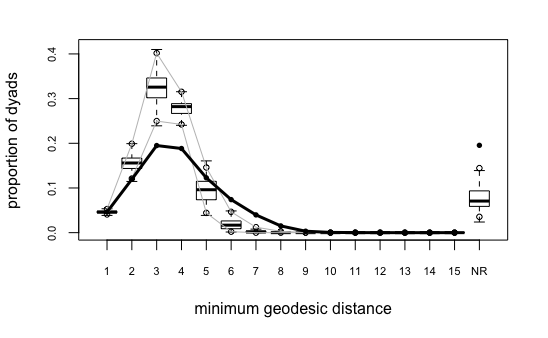

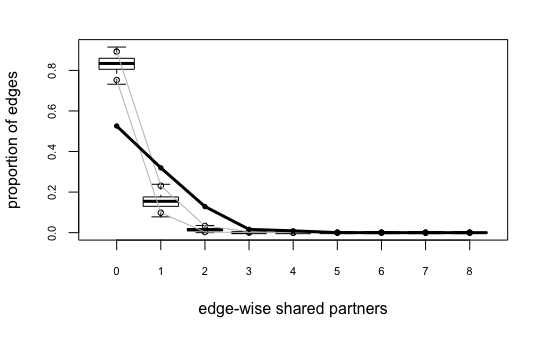

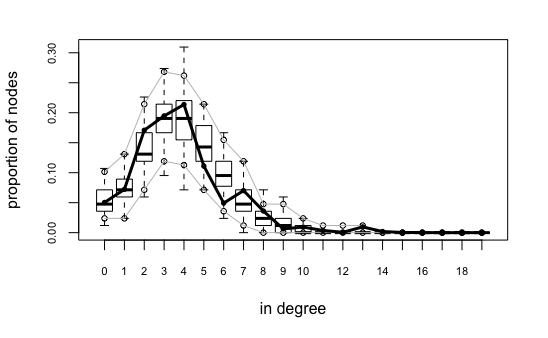


**Figure D. Goodness of fit plots for interactive fall offering.**
